# Novel *GALT* variations and mutation spectrum in the Korean population with decreased galactose-1-phosphate uridyltransferase activity

**DOI:** 10.1186/s12881-014-0094-5

**Published:** 2014-08-15

**Authors:** Rihwa Choi, Kyoung Il Jo, Dae-Hyun Ko, Dong Hwan Lee, Junghan Song, Dong-Kyu Jin, Chang-Seok Ki, Soo-Youn Lee, Jong-Won Kim, Yong-Wha Lee, Hyung-Doo Park

**Affiliations:** 1 Department of Laboratory Medicine and Genetics, Samsung Medical Center, Sungkyunkwan University School of Medicine, 81 Irwon-ro, Gangnam-gu, Seoul 135-710, Republic of Korea; 2 Department of Laboratory Medicine and Genetics, Soonchunhyang University Bucheon Hospital, Soonchunhyang University College of Medicine, Bucheon, Republic of Korea; 3 Department of Laboratory Medicine, Asan Medical Center, University of Ulsan College of Medicine, Seoul, Republic of Korea; 4 Department of Pediatrics, Soonchunhyang University Hospital, Soonchunhyang University College of Medicine, Seoul, Republic of Korea; 5 Department of Laboratory Medicine, Seoul National University Bundang Hospital, Seoul National University College of Medicine, Seongnam, Republic of Korea; 6 Department of Pediatrics, Samsung Medical Center, Sungkyunkwan University School of Medicine, Seoul, Republic of Korea

**Keywords:** Galactosemia, Galactose-1-phosphate uridyltransferase, GALT, Metabolic disease, Mutation

## Abstract

**Background:**

Classic galactosemia (OMIM #230400) is an autosomal recessive metabolic disorder caused by a deficiency of the galactose-1-phosphate uridyltransferase (GALT, EC2.7.7.12) protein due to mutations in the *GALT* gene. The aim of this study was to provide a comprehensive and updated mutation spectrum of *GALT* in a Korean population.

**Methods:**

Thirteen unrelated patients screened positive for galactosemia in a newborn screening program were included in this study. They showed a reduced GALT enzyme activity in red blood cells. Direct sequencing of the *GALT* gene and *in silico* analyses were done to evaluate the impact of novel variations upon GALT enzyme activity. We also reviewed previous reports for *GALT* mutations in Koreans.

**Results:**

We identified six novel likely pathogenic variations including three missense (p.Ala101Asp, p.Tyr165His, and p.Pro257Thr), one small deletion/insertion [c.826_827delinsAA (p.Ala276Asn)], one frameshift (p.Asn96Serfs*5), and one splicing (c.378-1G > C) likely pathogenic variations. The most frequent variation was the Duarte variant (c.940A > G, 35.3%), followed by c.507G > C (p.Gln169His, 9.6%), among 34 Korean patients. Other mutations were widely scattered. None of the eight common mutations used for targeted mutation analysis in Western countries including p.Gln188Arg, p.Ser135Leu, p.Lys285Asn, p.Leu195Pro, p.Tyr209Cys, p.Phe171Ser, c.253-2A > G, and a 5 kb deletion, had been found in Koreans until this study.

**Conclusions:**

Considering the mutation spectrum in Koreans, direct sequence analysis of entire *GALT* exons is recommended for accurate diagnosis. The mutations responsible for GALT deficiency in the Korean population were clearly different from those of other populations.

## Background

Classical galactosemia (OMIM #230400) is an autosomal recessive inborn error of metabolism caused by a deficiency of the galactose-1-phosphate uridyltransferase (GALT, EC2.7.7.12) protein, which is encoded by the *GALT* gene. *GALT* maps to chromosome 9p13 and includes 11 exons that span about 4 kb of genomic DNA [1]. Galactosemia is a potentially fatal disease that manifests within the first week of life with poor feeding, jaundice, vomiting, liver dysfunction, increased bleeding tendency, and septicemia, leading to death if untreated [2]. It is well known that early detection of galactosemia and management with dietary restriction of galactose can prevent infants from a deteriorating state and leads to rapid clinical improvement. Many patients develop long-term complications, with a broad range of clinical symptoms such as cataracts, speech defects, poor growth, poor intellectual function, neurologic deficits, and premature ovarian insufficiency [3]. Many countries have implemented newborn screening programs in order to detect and manage the disease earlier [4]. A wide range of galactosemia frequency was estimated in Western countries (1:14,000 to 1:80,000) [4],[5] and much lower frequency reported in Asian populations [6],[7]. To date, more than 230 mutations responsible for galactosemia have been identified and updated in the *GALT* gene databases [8],[9]. Eight common *GALT* mutations for targeted mutation analysis have been introduced in Western countries, which include p.Gln188Arg, p.Ser135Leu, p.Lys285Asn, p.Leu195Pro, p.Tyr209Cys, p.Phe171Ser, c.253-2A > G, and a 5 kb deletion [10]-[12]. However, none of these mutations have been reported in Asian populations. Since there is a wide range of mutations among different ethnic background, genetic analysis is needed to understand the mutation spectrum of *GALT* in the Korean population. In Korea, a nationwide newborn screening program for galactosemia, first introduced in 1991, is now performed on more than 90% of Korean newborns [13]. Only 21 Korean patients with GALT-deficient galactosemia have been reported, which suggests a low prevalence [14],[15]. The aim of this study was to characterize and update the spectrum of *GALT* gene variations in Korean patients using direct sequence analysis and to produce a comprehensive literature review of *GALT* mutations in the Korean population.

## Methods

### Patients

Thirteen unrelated Korean children with abnormal newborn screening results for galactosemia and reduced GALT activities between April 2010 and April 2013 from two institutions (Samsung Medical Center and Soonchunhyang University Hospital) were included in this study. Ninety-six healthy subjects were also included as controls in order to exclude common polymorphisms for six novel variations. Additionally, a comprehensive review of the literature with previously reported *GALT* mutations in Koreans was conducted. This research was approved by the Samsung Medical Center Institutional Review Board and written informed consent was obtained from all subjects and/or their parents whenever possible.

### GALT enzyme activity in red blood cells

GALT enzyme activity in red blood cells was measured by an established radiometric assay [16] and was expressed in μmol/hour (hr)/g hemoglobin (Hb). The reference range of GALT enzyme activity is from 20 to 35 μmol/hr/g Hb.

### DNA amplification and direct sequence analysis

Human genomic DNA was prepared from frozen white blood cells using a Wizard genomic DNA purification kit (Promega, Madison, WI, USA) according to the manufacturer’s recommendations. All 11 exons and the flanking regions of the *GALT* gene were amplified by polymerase chain reaction (PCR) using primers designed by the authors (sequences available upon request) and a thermal cycler (Model 970; Applied Biosystems, Foster City, CA, USA). Direct sequencing of the DNA was performed using the ABI Prism 3100 Genetic Analyzer (Applied Biosystems, Foster City, CA, USA) with the BigDye Terminator Cycle Sequencing-Ready Reaction Kit (Applied Biosystems Foster City, CA, USA). Nucleotides are numbered from the first adenine of the ATG translation initiation codon in the *GALT* cDNA Reference Sequence NM_000155.2.

### Pathologic predictions of novel substitution variants

We performed *in silico* analyses to identify the mutational status of a novel exonic variation using two different servers. PolyPhen-2 prediction was used to identify the functional effect of human non-synonymous single nucleotide polymorphisms (http://genetics.bwh.harvard.edu/pph2/) and the second prediction program was SIFT (Sorting Intolerant From Tolerant, http://sift.jcvi.org/). “Damaging change” in PolyPhen-2 and “not tolerated” in SIFT both mean that the missense variation may be a mutation rather than a polymorphism. We also used the Human Splicing Finder (http://www.umd.be/HSF/) to predict any difference in splicing between the novel mutants and their respective wild-type reference sequences to estimate the mutation status of novel substitution variants. If *in silico* analyses predicted the novel variants to be likely pathogenic, then the novel variations are defined as likely pathogenic variations rather than detrimental mutations because expression studies were not performed.

### Genetic analysis of normal individuals

The tests targeted the six novel sequence variations revealed in this study and were conducted on 192 alleles from 96 healthy adult individuals without any clinical symptoms of galactosemia. All genetic analyses were done using direct sequence analysis, as explained above.

### Statistical tests

We applied the Kruskal-Wallis test with post-hoc analysis for comparison of patients with different variant alleles for the *GALT* gene including the Duarte variant using the MedCalc program for Windows, version 12.2.1 (MedCalc Software, Mariakerke, Belgium). A *p* value of less than 0.05 was considered significant.

## Results

### Demographics of GALT-deficient galactosemia

During the 3-year study period, 13 unrelated patients with GALT deficiency (six males and seven females) were included in this study. The median age at molecular diagnosis in the 13 Korean galactosemia subjects was 2.9 months (range, 1 month – 23 months). All but one had no significant clinical symptoms or signs of galactosemia. Only one patient had jaundice and cataracts whose age at molecular diagnosis was 23 months. All of the children with abnormal newborn screening results for galactosemia and reduced GALT activities started treatment with dietary galactose restriction.

### Sequence variations in the GALT gene in 13 Korean galactosemia patients

All 13 patients with reduced GALT activity had various sequence variations in the *GALT* gene. A total of 20 variant alleles (76.9%) were identified, corresponding to 12 different variations. We identified seven alleles with five known pathogenic mutant alleles, seven alleles with six novel likely pathogenic variant alleles, and six alleles with the Duarte variant allele. The five known mutations included four missense mutations [c.346C > A (p.Leu116Ile), c.602G > A (p.Arg201His), c.998G > A (p.Arg333Gln), and c.1087G > A (p.Glu363Lys)] and one intronic mutation (c.821-7A > G), which causes a splicing aberration (exon 9 deletion). The six novel likely pathogenic variations consisted of three missense variations [c.302C > A (p.Ala101Asp), c.493 T > C (p.Tyr165His), and c.769C > A (p.Pro257Thr)], one small deletion/insertion [c.826_827delinsAA (p.Ala276Asn)], one frameshift [c.286_299delGACAACGACTTCCC (p.Asp96Serfs*5)], and one splicing (c.378-1G > C) variation. Five patients had the Duarte variant allele (c.940A > G) and one was homozygous. Individual characteristics of the *GALT* genotype in the 13 patients used in this study are summarized in Table 1.

**Table 1:** **Individual characteristics of the** ***GALT*** **genotype in 13 Korean patients with decreased GALT enzyme activity**

**Case no.**	**Age (month)**	**Sex**	**Location**	Nucleotide change*	**Amino acid change**	**GALT activity**^**†**^**(μmol/h/g Hb)**	**Category**^**‡**^
1	24.5	M	Exon 3	c.286_299delGACAACGACTTCCC	p.Asp96Serfs*5	< 0.1	G/G
			IVS 4	c.378-1G > C	p.?		
2	1.8	F	Exon 3	c.286_299delGACAACGACTTCCC	p.Asp96Serfs*5	7.3	G/G
			IVS 8	c.821-7A > G	Exon 9 deletion		
3	2.3	F	Exon 5	c.493 T > C	p.Tyr165His	6.3	G/G
			Exon 10	c.998G > A	p.Arg333Gln		
4	1.7	M	Exon 10	c.998G > A	p.Arg333Gln	1.8	G/G
			Exon 10	c.998G > A	p.Arg333Gln		
5	1.1	F	Exon 3	c.302C > A	p.Ala101Asp	6.2	G/D
			Exon 10	c.940A > G	p.Asn314Asp^§^		
6	2.9	M	Exon 9	c.826_827delinsAA	p.Ala276Asn	5.9	G/D
			Exon 10	c.940A > G	p.Asn314Asp^§^		
7	3.1	M	Exon 4	c.346C > A	p.Leu116Ile	15.6	G/N
8	2.2	F	Exon 7	c.602G > A	p.Arg201His	14.5	G/N
9	3.1	M	Exon 8	c.769C > A	p.Pro257Thr	11.6	G/N
10	2.9	F	Exon 11	c.1087G > A	p.Glu363Lys	16.4	G/N
11	3.0	F	Exon 10	c.940A > G	p.Asn314Asp^§^	12.5	D/D
12	3.5	F	Exon 10	c.940A > G	p.Asn314Asp^§^	12.6	D/N
13	5.2	M	Exon 10	c.940A > G	p.Asn314Asp^§^	16.1	D/N

*GALT* galactose-1-phosphate uridyltransferase.

*Nucleotide numbering was based on the GALT cDNA sequence (RefSeq NM_000155.2) with nucleotide +1 being the A of the first ATG translation initiation codon.

^†^The reference range of GALT enzyme activity was 20–35 μmol/h/g Hb for normal healthy individuals and the limit of quantitation was 0.1 μmol/h/g Hb.

^‡^D, Duarte variant; G, allele for galactosemia including novel likely pathogenic variant; N, normal allele.

^§^Duarte variant.

### Structural and functional effects of novel variations

*In silico* analyses for four novel nucleotide substitutions and one novel in frame deletion/insertion mutation were predicted using PolyPhen-2 and SIFT. The possible mis-splicing effect of exonic or intronic variations predicted using Human Splice Finder is summarized in Table 2. Three novel variations of c.302C > A (p.Ala101Asp), c.796C > A (p.Pro257Thr), and c.826_827delinsAA (p.Ala276Asn) were predicted as mutations by PolyPhen-2 and SIFT. The novel variation of c.493 T > C (p.Tyr165His) was predicted to be a polymorphism by both PolyPhen-2 and SIFT. However, it was predicted to activate a cryptic splice site of an enhancer motif causing aberrant splicing according to the Human Splice Finder matrix. The c.493 T > C variation was observed in one patient who was a compound heterozygote for the previously reported pathogenic mutation, c.998G > A (p.Arg333Gln), and the patient had 6.3 μmol/h/g Hb of GALT activity, suggesting that this variation might be pathogenic.

**Table 2:** Predicted effects of five novel variations (four exonic and one intronic variations) in 13 Korean patients with decreased GALT activity

**Location**	**Sequence variation**	**Amino acid change**	**Predicted effect**					GALT activity in patients*(μmol/hr/g Hb)
			**PolyPhen-2**		**SIFT**	**Splicing effect**	**Affected motif**	
			**Overall stability**	**Score**				
Exon 3	c.302C > A	p.Ala101Asp	Probably damaging	0.998	Not tolerated	Activation of a cryptic splice site	Enhancer motif	6.2
Exon 5	c.493 T > C	p.Tyr165His	Benign	0.440	Tolerated	Activation of a cryptic splice site	Enhancer motif	6.3
Exon 8	c.769C > A	p.Pro257Thr	Probably damaging	0.998	Not tolerated	No effect	No effect	11.6
Exon 9	c.826_827delinsAA	p.Ala276Asn	Probably damaging	0.995	Not tolerated	No effect	No effect	5.9
IVS4	c.378-1G > C	NA	NA	NA	NA	Activation of a cryptic splice site	Enhancer motif	<0.1
						Disruption of a natural motif	Silencer motif	

*NA* not applicable or not analyzed.

*The reference range of GALT enzyme activity was 20–35 μmol/h/g Hb for normal healthy individuals and the limit of quantitation was 0.1 μmol/h/g Hb.

The possible mis-splicing effect of silent exonic or intronic variations located outside of the canonical splicing signals, AG or GT, also revealed that c.302C > A (p.Ala101Asp) was predicted to activate a cryptic splice site of an enhancer motif. This variation was detected in one patient who was a compound heterozygote for the Duarte variant (D/G galactosemia) with 6.2 μmol/h/g Hb of GALT activity, which supports the hypothesis that this variation may be pathogenic rather than benign. The intronic variation of c.378-1G > C was predicted to impact the splice site through both activation of a cryptic splice site of an enhancer motif and disruption of a natural silencer motif. This variation was identified in one patient heterozygous for the other novel likely pathogenic variant allele [c.286_299delGACAACGACTTCCC (p. Asp96Serfs*5)] with GALT enzymatic activity below the quantitation limit of 0.1 μmol/h/g Hb. Also, another patient with 7.3 μmol/h/g Hb of GALT activity, was compound heterozygous for c.286_299delGACAACGACTTCCC and c.821-7A > G. A known mutant allele of c.821-7A > G was reported to cause an exon 9 deletion. In addition, six novel variations were absent in the 192 alleles from healthy individuals.

An intronic variation of c.82 + 20_82 + 60del was observed in one patient who was compound heterozygous for two other novel likely pathogenic variant alleles, c.286_299delGACAACGACTTCCC and c.378-1G > C, and had no GALT enzyme activity (<0.1 μmol/h/g Hb), which suggests that this variation is a polymorphism without significant enzymatic effect.

### Mutation spectrum of the GALT gene in Korean patients

Table 3 summarizes all the variations in the *GALT* gene, including previously published data in Korea and this study. Thirty-four Korean patients with GALT deficiency galactosemia were recruited and a total of 51 variant alleles were identified (75.0%, 51/68 alleles). The most frequent nucleotide substitution was the Duarte variant (c.940A > G, n = 18, 35.3%), followed by c.507G > C (p.Gln169His, n = 5, 9.8%). Among the 34 Korean patients, eight (23.5%) had classic galactosemia with two pathogenic variant alleles, eight (23.5%) were compound heterozygous for a Duarte variant and a pathogenic variant allele, nine (26.5%) were heterozygous for only one pathogenic variant allele, eight (23.5%) were heterozygous for one Duarte variant allele, and one (2.9%) was homozygous for the Duarte variant allele. The *GALT* mutational spectrum is comprised of 18 distinct variations that are widely scattered. Most of the putative disease-causing variations were missense variations (n = 11, 61.1%), with the other variants being small deletion/insertions (n = 2, 11.1%), exonic or intronic variations that could affect splicing (n = 4, 22.2%) and silent exonic variations (n = 1, 5.6%).

**Table 3:** Genetic spectrum and allele frequency of likely pathogenic GALT variations in 34 Korean GALT-deficient galactosemia patients

**Location**	**Nucleotide change**	**Amino acid change**	**Allele number**	**Previously reported in Koreans**
Missense mutation				
Exon 2	c.92A > G	p.His31Arg	1	Ko et al. [14]
Exon 3	c.302C > A	p.Ala101Asp	1	This study, novel
Exon 4	c.346C > A	p.Leu116Ile	2	Ko et al. [14], this study
Exon 5	c.493 T > C	p.Tyr165His	1	This study, novel
Exon 5	c.507G > C	p.Gln169His	5	Ko et al. [14], Lee et al. [15]
Exon 6	c.557A > C	p.His186Pro	3	Ko et al. [14]
Exon 7	c.602G > A	p.Arg201His	2	Ko et al. [14], this study
Exon 8	c.769C > A	p.Pro257Thr	1	This study, novel
Exon 10	c.940A > G	p.Asn314Asp*	18	Ko et al. [14], this study
Exon 10	c.998G > A	p.Arg333Gln	3	This study^†^
Exon 11	c.1087G > A	p.Glu363Lys	2	Lee et al. [15], this study
Small deletion/insertion leading to frameshift				
Exon 3	c.286_299delGACAACGACTTCCC	p.Asp96Serfs*5	2	This study, novel
Small deletion/insertion (in-frame)				
Exon 9	c.826_827delinsAA	p.Ala276Asn	1	This study, novel
Silent exonic variation				
Exon 10	c.999G > A	p.Arg333=	1	Ko et al. [14]
Splicing aberration				
IVS 2	c.252 + 1G > A	Exon 2 deletion	3	Lee et al. [15]
IVS 6	c.565-2A > G	Exon 7 deletion	1	Ko et al. [14]
IVS 8	c.821-7A > G	Exon 9 deletion	3	Ko et al. [14], this study
Predicted to cause splicing aberration				
IVS 4	c.378-1G > C	p.?	1	This study, novel
Considered as a rare polymorphism				
IVS 1	c.82 + 20_82 + 60del	p.?	1	This study, novel^‡^

*Duarte variant.

^†^Although this variation has not been previously reported in Koreans, it is a known pathogenic mutation identified in the Japanese population and reported by Hirokawa et al. [6].

^‡^This variation was found in a patient who was compound heterozygous for c.286_299delGACAACGACTTCCC and c.378-1G > C.

**Figure 1:**
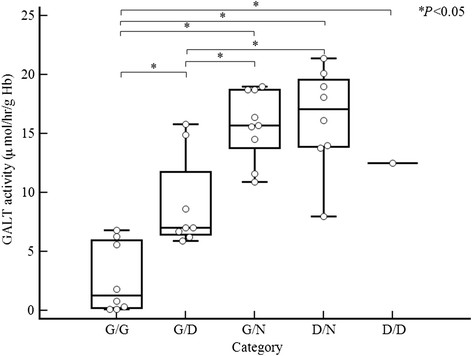
**GALT enzyme activities among different categories of GALT biochemical phenotypes in 34 Korean patients.** The bars in the boxes represent the median values, and the areas above and below the bars in the boxes represent the upper and lower quartiles, respectively. The lines extending below and above the boxes represent the lowest and highest values, respectively, and the circles represent individual data. *P* value was calculated by the comparison among five different groups of patients with GALT biochemical phenotypes by the Kruskal-Wallis test with post-hoc analysis (D, Duarte variant; G, mutant allele for galactosemia including novel likely pathogenic variant; N, normal allele). It is of note that although there were differences in GALT activity between the G/G and D/G patient groups, the GALT enzyme activities of individual patients were varied with overlapping distributions.

GALT activities of all cases including our data are summarized in Figure 1. The GALT enzyme activities of patients varied according to genotype with overlapping distributions: mean 3.9 μmol/h/g Hb for classic galactosemia patients (range, < 0.1–7.3), mean 6.1 μmol/h/g Hb for compound heterozygotes of Duarte and classic variants (range, 5.9–6.2), 12.5 μmol/h/g Hb for Duarte homozygotes, mean 14.5 μmol/h/g Hb for carriers of classic mutant alleles (range, 11.6–16.4), and mean 14.4 μmol/h/g Hb for carriers of Duarte variant alleles (range, 12.6–16.1). GALT activity of classic galactosemia patients was significantly different from other groups with different variant alleles (*p* < 0.05). The differences of GALT activities between carriers of classic variant alleles and carriers of the Duarte variant allele were not significantly different (*p* > 0.05). Because only one patient was homozygous for the Duarte variant allele, it was difficult to compare GALT activities among different groups with variant alleles and there were no significant differences from other groups of patients with variant alleles except for the group of classic galactosemia patients (*p* > 0.05).

## Discussion

Among 34 Korean patients with *GALT* mutations including likely pathogenic variants, the most common type of disease-causing mutation was a missense mutation (61.1%) followed by a splicing mutation (22.2%). The most common mutation except for the Duarte variant was c.507G > C (p.Gln169His) and it was found in five out of 51 variant alleles (9.8%). Other mutations previously found in the Korean population were widely scattered in our patients and no other specific mutations were found to be prevalent at a significant frequency, which makes targeted mutation analysis difficult.

Our study also reaffirmed that measurement of GALT enzyme activity was not enough to predict the genotypes and to make a decision for treatment in some patients because of the lack of correlation between genotype and phenotype [17],[18]. For the galactosemia variant, the D/G phenotype of GALT activity in which at least one mutation is the c.940A > G (p.Asn314Asp) allele with the enzyme activity between 5% and 50% of normal control values and with which there is no evidence for hepatocellular damage, cataract, or developmental delays during infancy, the need to restrict dietary galactose is uncertain [19],[20]. In our study, there were eight patients with the D/G GALT biochemical phenotype among 34 GALT-deficient galactosemia patients, and these patients had an enzyme activity of 5.9–15.8 μmol/h/g Hb (median 7.0 μmol/h/g Hb), corresponding to 21.5–57.5% of the mean control value. Although there were differences in GALT activity between the G/G and D/G patient groups, the GALT enzyme activities of individual patients were varied with overlapping distributions. It is reasonable and important to distinguish D/G galactosemia from G/G classic galactosemia using a molecular diagnostic test prior to making any treatment decisions. We report the first Korean patient with two Duarte variants who is in the homozygous state of the Duarte variant allele (D/D). Her GALT activity was 12.5 μmol/h/g Hb corresponding to 35.7–62.5% of normal control values (20–35 μmol/h/g Hb), which is consistent with previously reported data [21].

The incidence of GALT galactosemia varies, ranging from 1 in 23,000–40,000 in Europe [17],[22] to 1 in 50,000 in the United States [10]. It has been reported that the frequency of GALT galactosemia has decreased in continental Europe, moving through populations in an eastern and southern direction [17]. Using haplotype analysis in conjunction with a measure of genetic diversity and linkage disequilibrium, it was found that p.Gln188Arg arose in central Europe within the last 20,000 years, possibly due to population expansion during the re-colonization of Europe by *Homo sapiens* in the Mesolithic Age. p.Lys285Asn is a younger mutation that originated in Eastern Europe and is probably more geographically restricted since it arose after all major European population expansions [23]. The Duarte variant, c.940A > G (p.Asn314Asp), was found to be an ancient mutation that originated before the expansion of *Homo sapiens* out of Africa [10],[23]. In Asian countries, the incidences of classic galactosemia is reported to be much lower than in Western countries, ranging from 1 in 1,000,000 in Japan to 1 in 500,000 in China [6].

In Korea, the nationwide newborn screening for galactosemia was first introduced in 1991. From 1997 to 2005, it was stopped due to budget concerns and cost-effectiveness of the newborn screening program and then resumed in 2006 [24]. According to the annual report of the Ministry of Health and Welfare of Korea, about 80.4% of newborns were screened by the nationwide newborn screening program from 1991 to 2007. After 2006, the incidence of galactosemia has increased from 1:100,000 in 2006 to around 1:15,000 in 2012, and prevalence of galactosemia in Korea was calculated to be between 1:30,000 and 1:35,000 from 2005 to 2012 [13],[25]. However, the exact incidence and prevalence of classic galactosemia is not yet known. Although it is very difficult to evaluate the accurate prevalence of classic galactosemia in the Korean population, some previous reports have suggested a lower prevalence in Koreans compared to Western countries [13]-[15],[24]. Ko et al. reported only one classic galactosemia patient in 18 patients with reduced GALT activities [14], and Lee et al. reported three classic galactosemia patients during the 11-year study period [15], which suggested a low prevalence of classic galactosemia in the Korean population. However, during the 3-year period in this study, which was relatively shorter than in previous studies, four classic galactosemia patients were diagnosed, which suggests that the prevalence of classic galactosemia in the Korean population may be increasing. One cause for the increase in galactosemia incidence could be due to a different panel of populations screened. Another reason attributing to the higher prevalence observed may be due to different methods used more recently with respect to old methods. Lastly, other unknown variables that have been taken into account to explain the increasing of prevalence. In Korea, the accreditation and inspection program by the Planned Population Federation of Korea for the proper quality assurance of clinical laboratory performing newborn screening tests has been settled since 2008 which could also affect the increasing prevalence of galactosemia [4],[25]. Further studies are needed to evaluate the accurate prevalence of classic galactosemia combined with comprehensive monitoring program.

To date, more than 230 mutations have been identified in the *GALT* gene databases [8],[9]. Eight common *GALT* mutations for targeted mutation analysis of p.Gln188Arg, p.Ser135Leu, p.Lys285Asn, p.Leu195Pro, p.Tyr209Cys, p.Phe171Ser, c.253-2A > G, and a 5 kb deletion have been introduced as a cost-effective diagnostic strategy in Western countries [10]-[12]. Also, other diagnostic methods such as matrix assisted laser desorption/ionization time-of-flight mass spectrometry, PCR using an amplification refractory mutation system, and multiplexed single nucleotide extension techniques have also been introduced for both population-based screening and diagnosis in patients [4],[18],[26]-[28]. However, none of the above eight common mutations have been found in the Korean population as well as another East Asian populations including Chinese and Japanese [6],[7],[14],[15],[29]. These findings strongly indicate that the Asian *GALT* mutations are clearly different from the European mutations because of the genetic divergence of *Homo sapiens*[6],[17],[23]. Although further evaluation is needed to confirm the origin and the exact effect of the six novel likely pathogenic variations identified in this study, our work suggests that these variants could be young mutations that may only be found in those of Asian ethnicity. Taking into account of all these findings, it is reasonable to suggest that the mutation databases in the Asian population be separated or regarded as a distinct category from those of Western countries for the best diagnostic approach and for development and application of therapeutic approaches in Asian patients with GALT-deficient galactosemia.

It is possible that the difference in the prevalence of classic galactosemia between countries is due to technical issues with the population-based newborn screening programs rather than true population differences [4]. Differences in approach or sensitivity of the methods used to detect GALT deficiency, or in defined cut-off levels, may leave some heterozygotes and/or Duarte variants included in some population estimates but not others [4]. For example, Africa has an estimated prevalence of 1:14,000, which is based on a population-based screen of cord blood samples for the p.Ser135Leu *GALT* mutation and accounts for > 90% of all classic galactosemia cases in this population [30]. The increasing detection rate of galactosemia in the Korean population should be further evaluated with a focus on the screening procedures.

## Conclusions

In summary, we identified six novel likely pathogenic variations and expanded the genetic spectrum of *GALT* variations in Koreans. The mutations responsible for GALT deficiency in the Korean population were clearly different from those of other populations, and common mutations with high frequency in Western countries are not found in Koreans. *GALT* mutations are very widely scattered along the entire length of the gene, so targeted mutational analysis for screening or diagnosis is not appropriate.

### Compliance with ethics guideline

The content of this article represents original work that has not been previously published nor in consideration of publication elsewhere. The manuscript has been read and approved for submission to the journal by all contributing authors.

## Competing interests

The authors declare that they have no competing interests.

## Authors’ contributions

RC analyzed genetic data and was responsible for drafting the manuscript. KJ investigated clinical and biochemical information of the patients. DK analyzed biochemical and molecular genetic data. DL was supplier of samples and clinical data. JS was supplier of samples and clinical data. DJ was supplier of samples and clinical data. CK carried out the PCR and sequencing analysis. SL carried out the biochemical analysis. JK helped to draft the manuscript. YL revised the manuscript for important intellectual content. HP coordinated whole research and helped to revise the manuscript. All authors read and approved the final manuscript.

## References

[B1] Leslie ND Immerman EB Flach JE Florez M Fridovich-Keil JL Elsas LJ The human galactose-1-phosphate uridyltransferase gene Genomics 1992 14 2 474 480 10.1016/S0888-7543(05)80244-7 1427861

[B2] Bosch AM Classical galactosaemia revisited J Inherit Metab Dis 2006 29 4 516 525 10.1007/s10545-006-0382-0 16838075

[B3] Waisbren SE Potter NL Gordon CM Green RC Greenstein P Gubbels CS Rubio-Gozalbo E Schomer D Welt C Anastasoaie V D’Anna K Gentile J Guo CY Hecht L Jackson R Jansma BM Li Y Lip V Miller DT Murray M Power L Quinn N Rohr F Shen Y Skinder-Meredith A Timmers I Tunick R Wessel A Wu BL Levy H The adult galactosemic phenotype J Inherit Metab Dis 2012 35 2 279 286 10.1007/s10545-011-9372-y 21779791 PMC3641771

[B4] Jumbo-Lucioni PP Garber K Kiel J Baric I Berry GT Bosch A Burlina A Chiesa A Pico ML Estrada SC Henderson H Leslie N Longo N Morris AA Ramirez-Farias C Schweitzer-Krantz S Silao CL Vela-Amieva M Waisbren S Fridovich-Keil JL Diversity of approaches to classic galactosemia around the world: a comparison of diagnosis, intervention, and outcomes J Inherit Metab Dis 2012 35 6 1037 1049 10.1007/s10545-012-9477-y 22450714 PMC3774053

[B5] Bosch AM Ijlst L Oostheim W Mulders J Bakker HD Wijburg FA Wanders RJ Waterham HR Identification of novel mutations in classical galactosemia Hum Mutat 2005 25 5 502 10.1002/humu.9330 15841485

[B6] Hirokawa H Okano Y Asada M Fujimoto A Suyama I Isshiki G Molecular basis for phenotypic heterogeneity in galactosaemia: prediction of clinical phenotype from genotype in Japanese patients Eur J Hum Genet 1999 7 7 757 764 10.1038/sj.ejhg.5200368 10573007

[B7] Cheung KL Tang NL Hsiao KJ Law LK Wong W Ng PC Pang CP Applegarth DA Fok TF Hjelm NM Classical galactosaemia in Chinese: a case report and review of disease incidence J Paediatr Child Health 1999 35 4 399 400 10.1046/j.1440-1754.1999.00373.x 10457302

[B8] Calderon FR Phansalkar AR Crockett DK Miller M Mao R Mutation database for the galactose-1-phosphate uridyltransferase (GALT) gene Hum Mutat 2007 28 10 939 943 10.1002/humu.20544 17486650

[B9] http://www.hgmd.org/**The human gene mutation database.**.

[B10] Suzuki M West C Beutler E Large-scale molecular screening for galactosemia alleles in a pan-ethnic population Hum Genet 2001 109 2 210 215 10.1007/s004390100552 11511927

[B11] Elsas LJ 2nd Lai K The molecular biology of galactosemia Genet Med 1998 1 1 40 48 10.1097/00125817-199811000-00009 11261429

[B12] Coffee B Hjelm LN DeLorenzo A Courtney EM Yu C Muralidharan K Characterization of an unusual deletion of the galactose-1-phosphate uridyl transferase (GALT) gene Genet Med 2006 8 10 635 640 10.1097/01.gim.0000237720.78475.fb 17079880

[B13] Lee DH The prevalence of pediatric endocrine and metabolic diseases in Korea Korean J Pediatr 2008 51 6 559 563 10.3345/kjp.2008.51.6.559

[B14] Ko DH Chang HE Song SH Park KU Kim JQ Kim MC Song YH Hong YH Lee DH Song J Molecular and biochemical characterization of the GALT gene in Korean patients with galactose-1-phosphate uridyltransferase deficiency Clin Chim Acta 2010 411 19–20 1506 1510 10.1016/j.cca.2010.06.008 20547145

[B15] Lee BH Cheon CK Kim JM Kang M Kim JH Yang SH Kim GH Choi JH Yoo HW Low prevalence of classical galactosemia in Korean population J Hum Genet 2011 56 1 94 96 10.1038/jhg.2010.152 21150919

[B16] Shin YS Hommes FA Galactose metabolites and disorders of galactose metabolism Techniques in Diagnostic Human Biochemical Genetics 1991 Wiley-Liss, New York 267 283

[B17] Tyfield L Reichardt J Fridovich-Keil J Croke DT Elsas LJ 2nd Strobl W Kozak L Coskun T Novelli G Okano Y Zekanowski C Shin Y Boleda MD Classical galactosemia and mutations at the galactose-1-phosphate uridyl transferase (GALT) gene Hum Mutat 1999 13 6 417 430 10.1002/(SICI)1098-1004(1999)13:6<417::AID-HUMU1>3.0.CO;2-0 10408771

[B18] Jama M Nelson L Pont-Kingdon G Mao R Lyon E Simultaneous amplification, detection, and analysis of common mutations in the galactose-1-phosphate uridyl transferase gene J Mol Diagn 2007 9 5 618 623 10.2353/jmoldx.2007.070027 17884932 PMC2049049

[B19] Ficicioglu C Thomas N Yager C Gallagher PR Hussa C Mattie A Day-Salvatore DL Forbes BJ Duarte (DG) galactosemia: a pilot study of biochemical and neurodevelopmental assessment in children detected by newborn screening Mol Genet Metab 2008 95 4 206 212 10.1016/j.ymgme.2008.09.005 18976948

[B20] Fernhoff PM Duarte galactosemia: how sweet is it? Clin Chem 2010 56 7 1045 1046 10.1373/clinchem.2010.147371 20489130

[B21] Lai K Langley SD Dembure PP Hjelm LN Elsas LJ 2nd Duarte allele impairs biostability of galactose-1-phosphate uridyltransferase in human lymphoblasts Hum Mutat 1998 11 1 28 38 10.1002/(SICI)1098-1004(1998)11:1<28::AID-HUMU5>3.0.CO;2-H 9450900

[B22] Murphy M McHugh B Tighe O Mayne P O’Neill C Naughten E Croke DT Genetic basis of transferase-deficient galactosaemia in Ireland and the population history of the Irish Travellers Eur J Hum Genet 1999 7 5 549 554 10.1038/sj.ejhg.5200327 10439960

[B23] Flanagan JM McMahon G Brendan Chia SH Fitzpatrick P Tighe O O’Neill C Briones P Gort L Kozak L Magee A Naughten E Radomyska B Schwartz M Shin JS Strobl WM Tyfield LA Waterham HR Russell H Bertorelle G Reichardt JK Mayne PD Croke DT The role of human demographic history in determining the distribution and frequency of transferase-deficient galactosaemia mutations Heredity 2010 104 2 148 154 10.1038/hdy.2009.84 19639008

[B24] Lee DH Newborn screening of inherited metabolic disease in Korea Korean J Pediatr 2006 49 11 1125 1139 10.3345/kjp.2006.49.11.1125

[B25] Analysis of Blood Sample Records for Neonatal Screening Test, External Quality Assessment and Field Assessment for Inborn Errors of Metabolism in Korea 2012 Welfare MoH, Seoul, Korea

[B26] Goldstein N Cohen Y Pode-Shakked B Sigalov E Vilensky B Peleg L Anikster Y The GALT rush: high carrier frequency of an unusual deletion mutation of the GALT gene in the Ashkenazi population Mol Genet Metabol 2011 102 2 157 160 10.1016/j.ymgme.2010.10.007 21059483

[B27] Barbouth D Slepak T Klapper H Lai K Elsas LJ Prevention of a molecular misdiagnosis in galactosemia Genet Med 2006 8 3 178 182 10.1097/01.gim.0000204019.54509.40 16540753

[B28] Mahmood U Imran M Naik SI Cheema HA Saeed A Arshad M Mahmood S Detection of common mutations in the GALT gene through ARMS Gene 2012 509 2 291 294 10.1016/j.gene.2012.08.010 22963887

[B29] Ashino J Okano Y Suyama I Yamazaki T Yoshino M Furuyama J Lin HC Reichardt JK Isshiki G Molecular characterization of galactosemia (type 1) mutations in Japanese Hum Mutat 1995 6 1 36 43 10.1002/humu.1380060108 7550229

[B30] Henderson H Leisegang F Brown R Eley B The clinical and molecular spectrum of galactosemia in patients from the Cape Town region of South Africa BMC Pediatr 2002 2 7 10.1186/1471-2431-2-7 12350230 PMC126267

